# BMP9 Crosstalk with the Hippo Pathway Regulates Endothelial Cell Matricellular and Chemokine Responses

**DOI:** 10.1371/journal.pone.0122892

**Published:** 2015-04-24

**Authors:** Kira Young, Eric Tweedie, Barbara Conley, Jacquelyn Ames, MaryLynn FitzSimons, Peter Brooks, Lucy Liaw, Calvin P. H. Vary

**Affiliations:** 1 Center for Molecular Medicine, Maine Medical Center Research Institute, Scarborough, Maine 04074, United States of America; 2 Graduate School of Biomedical Sciences and Engineering, University of Maine, Orono, Maine 04469, United States of America; Institute of Molecular and Cell Biology, Biopolis, UNITED STATES

## Abstract

Endoglin is a type III TGFβ auxiliary receptor that is upregulated in endothelial cells during angiogenesis and, when mutated in humans, results in the vascular disease hereditary hemorrhagic telangiectasia (HHT). Though endoglin has been implicated in cell adhesion, the underlying molecular mechanisms are still poorly understood. Here we show endoglin expression in endothelial cells regulates subcellular localization of zyxin in focal adhesions in response to BMP9. RNA knockdown of endoglin resulted in mislocalization of zyxin and altered formation of focal adhesions. The mechanotransduction role of focal adhesions and their ability to transmit regulatory signals through binding of the extracellular matrix are altered by endoglin deficiency. BMP/TGFβ transcription factors, SMADs, and zyxin have recently been implicated in a newly emerging signaling cascade, the Hippo pathway. The Hippo transcription coactivator, YAP1 (yes-associated protein 1), has been suggested to play a crucial role in mechanotransduction and cell-cell contact. Identification of BMP9-dependent nuclear localization of YAP1 in response to endoglin expression suggests a mechanism of crosstalk between the two pathways. Suppression of endoglin and YAP1 alters BMP9-dependent expression of YAP1 target genes CCN1 (cysteine-rich 61, CYR61) and CCN2 (connective tissue growth factor, CTGF) as well as the chemokine CCL2 (monocyte chemotactic protein 1, MCP-1). These results suggest a coordinate effect of endoglin deficiency on cell matrix remodeling and local inflammatory responses. Identification of a direct link between the Hippo pathway and endoglin may reveal novel mechanisms in the etiology of HHT.

## Introduction

Hereditary hemorrhagic telangiectasia (HHT) is a progressive vascular disease that affects as many as 1 in 5,000 people [1, 2]. Many familial variants of this autosomal dominant vascular dysplasia, also known as Osler-Weber-Rendu disease, have been identified worldwide. Diagnosed cases of HHT share the commonality of mutations in specific members of the transforming growth factor-beta (TGFβ) signaling pathway. The most frequent instances of HHT are caused by mutations that occur in the endoglin or ALK1 genes, referred to as HHT1 and HHT2, respectively. HHT is characterized by symptoms that include recurrent, severe nosebleeds, multiple small vascular malformations (telangiectasias) in the skin and various mucosa, and development of arteriovenous malformations (AVMs) in the lung [3, 4], liver [5], and brain [6]. AVMs establish a direct connection between veins and arteries, with a loss of the capillary bed intermediate, causing a shunting of blood [7–9]. The angiogenic signaling events resulting in vascular malformation initiation, location, and progression are still poorly understood.

Despite extensive study, endoglin’s biological function and the molecular mechanisms underlying the signaling consequences of its expression in endothelial cells (reviewed in [10, 11]), have yet to be fully elucidated. Endoglin (CD105) is a type III TGFβ coreceptor that associates with multiple TGFβ type I receptors (e.g., ALK1, ALK5) and type II receptors (e.g., TβRII) [12–14]. The endoglin null mouse phenotype is embryonic lethal by day 10.5 due to vascular defects, pointing to an essential role for endoglin in vascular development [15–17]. Endoglin expression increases in endothelial cells during angiogenesis [11], inflammation [18, 19], and the vascularization of tumors [20, 21]. Interestingly, a diagnosis of HHT, and hence, a decrease of endoglin expression, is correlated with improved cancer outcomes [22]. This may be due, in part, to the reduction in tumor stromal cell investment in the microenvironment [20]. However, it remains an important goal to better understand how endoglin expression, and endoglin deficiency in the case of HHT, alters downstream angiogenic signaling and vascular integrity.

Endoglin participates in canonical and non-canonical TGFβ signaling. When associated with TGFβ receptors, endoglin can bind several ligands including TGFβ, activins, and BMPs [12]. Endoglin has been shown to bind BMP9 independently of type I/II TGFβ receptors in endothelial cells [23–25]. This suggests endoglin levels may promote or repress signaling [13, 26–28] via distinct mechanisms that depend on the levels of endoglin, receptors, and ligands. Recent microarray and mass spectrometric analyses of endothelial cell BMP9-dependent responses implicate BMP9 in the regulation of chemokine signaling pathways such as SDF1/CXCR4 [18, 29] and monocyte chemoattractant protein (MCP-1/CCL2) [30] inflammation modulators, as well as extracellular matrix (ECM)-associated remodeling processes [29]. These insights suggest an unappreciated contribution by undiscovered BMP9 target proteins contributing to vessel integrity, extracellular matrix composition, and ECM-associated proteins.

In addition to its role as a coreceptor involved in the regulation of TGFβ superfamily signaling, endoglin may also impact endothelial cell behavior via regulation of cell adhesion. Cellular sites of focal adhesion formation provide mechanical linkage [31, 32] and cellular regulation by acting as a direct, dynamic connection between the cell and the ECM [33]. Integrins [34, 35] and zyxin [36–38] are among the molecules that comprise focal adhesions and that can broadly modulate receptor activity, which may also serve to integrate canonical TGFβ receptor signals [39–41]. Integrin β1 [42], zyxin [43], and the zyxin homolog, zyxin-related protein-1 [44], interact with endoglin [42, 43]. Interestingly, integrin ligands, including collagen [45], fibronectin [42], and other major ECM components [29] have all been shown to be directly regulated by endoglin expression and function, thus further establishing potential multi-level linkages between endoglin, focal adhesions, and the ECM.

Zyxin has been identified as a component of the newly emerging Hippo pathway [46]. Hippo signaling is regulated by cellular interactions with the ECM [47], potentially impacting cell chemotaxis, spreading, adhesion [48], and mechanotransduction sensing via zyxin [31]. The Hippo pathway downstream transcription coactivator, YAP1 (Yes kinase-associated protein 1, [49]), regulates the expression of matricellular CCN protein family members CTGF and CYR61 [50, 51]. BMP-Hippo crosstalk is suggested as CTGF is regulated by BMP signaling in non-endothelial cell types, including osteoblastogenesis [52] and diabetic neuropathy [53], as well as in mesenchymal stem cell osteogenesis [54]. This regulatory pathway links YAP1-dependent expression of CTGF and CYR61 to alterations in integrin and ECM component signaling [55].

Here we show that CTGF and CYR61 are BMP9-regulated target genes in endothelial cells. We expand upon the role endoglin plays in sites of focal adhesion and regulation of CTGF and CYR61 expression in response to BMP9. This role for BMP9 suggests novel endoglin-dependent interactions between BMP and Hippo pathway signaling and regulation of ECM metabolism. Finally, BMP9 treatment of endothelial cells coordinately alters their pattern of chemokine ligand and receptor expression [29]. We expand on this theme by showing that the CYR61- and CTGF-regulated chemokine, CCL2 [56, 57], is potently repressed by BMP9 while its receptor, CCR2, is upregulated. These results further develop the understanding of BMP9 and endoglin signaling effects in endothelial cells and provide new insights into the potential causes of HHT.

## Materials and Methods

### Cell culture

Human umbilical vein endothelial cells (HUVECs) were used for experiments at passage 2–7 and maintained in EGM-2 medium at 37°C and 5% CO_2_. Human primary endothelial cells and EGM-2 were purchased from Lonza Walkersville. EGM-2 medium consists of EMB-2 with 1% FBS and additives, including growth factors. Cells were treated with human recombinant BMP9 or TGFβ (R&D Systems) as indicated in the figure legends and text. FBS and additives were reduced to half the amount in culture conditions during ligand stimulations.

### Focal adhesion protein isolation

HUVECs, either shNT- or shENG-transduced, were plated in 10 cm fibronectin coated dishes (BD Biosciences) at 50% confluence for 24 hr. Cells were treated with BMP9 or TGFβ (5 ng/ml) for 24 hr. Cells were hypotonically shocked with low osmotic strength buffer (2.5 mM triethylamine, TEA, in water, pH = 7) as described previously [58]. Briefly, cells were removed from the plates using a Waterpik (setting “3”, Interplak dental water jet WJ6RW, Conair) to pulse 50 ml of PBS and protease inhibitors (Roche Diagnostics). The PBS buffer stream was held approximately 0.5 cm away and 90 degrees to the plates, this was done twice for 10 seconds each time. The PBS buffer (50 ml) was collected and reused for each dish in the each experimental set and collected for the total cell lysate fraction and Western blot analysis. Focal adhesions bound to the dish were rinsed thoroughly with PBS buffer (400 ml) with the Waterpik, and this fraction was scraped off plates with RIPA buffer (50 mM Tris-HCl, pH = 8, 150 mM NaCl, 1% NP-40, 0.5% sodium deoxycholate), 1% SDS, and sonicated for 15 seconds on ice using a Branson Sonifier 250 (duty cycle at 30% and power setting of 3). To permit comparison of different cellular fractions that don’t necessarily express common housekeeping proteins, total protein levels were determined by the BCA protein assay (ThermoScientific), and these values used to normalize loading volumes.

### Western blot analysis

For total cell lysate (TCL) protein preparation, cells were disrupted in RIPA lysis buffer (150 mM NaCl, 300 mM sucrose, 1% Triton X-100, 0.5% sodium deoxycholate, 50 mM Tris-HCl, pH = 7.5) containing protease (Roche Diagnostics) and phosphatase inhibitors (Calbiochem-EMD) as previously described [14]. The HUVEC cellular fractions were isolated according to the protocol in the Qproteome cell fractionation kit (Qiagen). For co-immunoprecipitation studies, cell culture plates were washed three times with ice-cold PBS and lysates were prepared using a modified RIPA buffer containing protease and phosphatase inhibitors as above. Mouse lung tissue was disrupted using an Omni homogenizer for 5 seconds at low speed followed by using a QIAshredder homogenizer. Following normalization of protein concentrations by BCA assay, Western blot analysis was performed with α-zyxin (Synaptic Systems), α-endoglin (BD Biosciences), α-β-actin (Sigma-Aldrich), α-YAP (Santa Cruz sc-101199), α-14-3-3ζ (GeneTex), α-CYR61 (Santa Cruz sc-8561), α-CTGF (Santa Cruz sc-14939), α-Integrin α5 (Santa Cruz sc-10729), and α-Integrin β1 (BD Transduction Laboratories Inc.) antibodies.

### RNA Extraction and Real Time Quantitative RT-PCR Analysis

RNA isolation and real-time quantitative RT-PCR (qRT-PCR) analyses were carried out essentially as described [29]. For these experiments, cells plated on fibronectin were harvested and washed, and their total RNA was isolated using RNeasy plus (QIAGEN). qRT-PCR reactions were run in triplicate on a BioRad iQ5 system. Primers used for quantitative RT-PCR and PCR analyses are listed below.

### qRT-PCR Primer sets

Human and mouse endoglin: 5’-AGCCCCACAAGTCTTGCAG-3’ and 5’-GCTAGTGGTATATGTCACCTCGC-3’. Human CTGF: 5’- CCAAACCGTGGTTGGGCCTG-3’ and 5’-GGCGCTCCACTCTGTGGTCT-3’. Mouse CTGF: 5’-ACCCAACTATGATGCGAGCC-3’ and 5’-GTAACTCGGGTGGAGATGCC-3’. Human CYR61: 5’-TTGAGCTGCTTGGCGCAGAC-3’ and 5’-ACAATGAGCTCCCGCATCGC-3’. Mouse CYR61: 5’-AGAGGCTTCCTGTCTTTGGC-3’ and 5’-CCAAGACGTGGTCTGAACGA-3’. Human CCL2: 5’-AGCAGCAAGTGTCCCAAAGA-3’ and 5’-TTTGCTTGTCCAGGTGGTCC-3’. Mouse CCL2: 5’-ATGCAGTTAACGCCCCACTC-3’ and 5’-GCTTCTTTGGGACACCTGCT-3’. Human and mouse CCR2: 5’-TCAGAAGCCTTTTTCACATAG-3’ and 5’-ACACTGGTTTTTGGAGTGGG-3’. β2-microglobulin and cyclophilin primers were used for normalization. Human β2-microglobulin; Forward: 5’-AAAGATGAGTATGCCTGCCG-3’, Reverse: 5’-CCTCCATGATGATGCTGCTTACA-3’. Mouse β2-microglobulin; Forward: 5'- CTGACCGGCCTGTATGCTAT-3', Reverse: 5'-CCGTTC-TTCAGCATTTGGAT -3'. Human cyclophilin; Forward: GAATAAGTTTGACTTGTGTTT; Reverse: CTAGGCATGGGAGGGAACA. Statistical significance is presented as the p value, with significance set to p = 0.05 or less.

### Lentiviral Transduction

Constructs expressing 21-nucleotide endoglin-specific short hairpin RNAs (shRNA) targeting human endoglin (shENG, shAlk1, shYAP) or nontargeting control (shNT, Sigma-Aldrich, SHC002) were used as described previously [29]. Human-targeting shYAP1 lentiviral shRNA was obtained from the Thermo Scientific RNAi consortium (TRCN0000107625). Constructs were packaged into lentivirus pseudotyped with the vesicular stomatitis virus glycoprotein. Transduction was performed by incubating cells with lentivirus, and stably transduced cells were subsequently used for studies. All cell lines were verified by morphology and mouse and human endoglin-specific PCR. They were certified mycoplasma-negative by PCR (Lonza), and primary cell cultures were used within the indicated passage numbers. Cells were transduced and selected using puromycin as described [29].

### Immunofluorescence and Confocal Microscopy

HUVECs were plated on 25-mm fibronectin-coated glass coverslips (BioCoat, BD Biosciences) at 40% confluence. Cells were allowed to adhere for 1 hr followed by 24 hr BMP9 treatment in EMB-2 (0.5% FBS, ½ the amount of growth factor additives in kit). Coverslips were washed twice with 1X PBS, fixed with 4% paraformaldehyde for 15 minutes and then washed twice with PBS and incubated in blocking solution (5% BSA, 0.1% Triton X-100, 0.1% Tween 20 in PBS) overnight at 4°C. Primary antibodies were diluted 1:200 in blocking solution and added to slides for a 1 hr incubation at room temperature. Coverslips were washed twice with 1X PBS before applying secondary antibody, diluted 1:1000 in blocking solution, and incubated for 30 minutes at room temperature. After a final 1X PBS wash, coverslips were mounted on glass slides using VECTASHIELD (Vector Laboratories) and observed using the LTCS-SP confocal system with an inverted DMIRBE microscope and a 100x objective (Leica). Antibodies used for immunofluorescence were: α-zyxin (Invitrogen), α-SMAD1/5 (Santa Cruz sc-6031-R), α-YAP (Cell Signaling), α-SMAD 2/3 (B&D Transduction), and α-CTGF (Santa Cruz sc-14939). Relative pixel intensity was measured in the cytoplasmic and nuclear areas of confocal immunofluorescence images using the FIJI version of ImageJ. Significance was determined using Student’s T test on six measurements on three separate cells per frame.

### Preparation of conditioned media

Conditioned medium (50 ml from 5 confluent 15-cm plates of shRNA random sequence control (NT) HUVECs or BMP9-treated HUVECs) was concentrated using an Amicon Ultracell 3 kDa centrifugal ultrafiltration cartridge as previously described [20, 29]. Following concentration, it was precipitated using 9 volumes of ethanol and stored at -80°C for 16 hr. The precipitate was collected by centrifugation and gently dried.

### Isotope-encoded affinity tag (ICAT) mass spectrometry

Precipitated cellular fractions and conditioned medium samples were dissolved in ICAT denaturing buffer containing the reducing agent trichloroethylphosphine. Following reaction with heavy and light ICAT reagents (ABSciex), samples were digested with trypsin as previously described [20, 29]. After desalting (PepClean C18 column, Pierce), tryptic peptides were separated using a linear water/acetonitrile gradient (0.1% formic acid) on a Acclaim PepMap reversed-phase capillary column (3 μm, C18, 100 Angstrom pore size, 75 μm ID x 15 cm, 15 μm tip; ThermoFisher) with an inline PepMap 100 precolumn (C18, 300 μm ID x 5 mm; LC Packings) as a loading column and infused onto a quadrupole-time-of-flight mass spectrometer (QSTAR, ABSciex) as described [20, 29]. Identification and quantitation of heavy isotope-tagged (BMP9-treated) versus light-isotope-tagged (control vehicle-treated) peptides were accomplished using ProteinPilot software (v4.5, ABSciex), with a mass tolerance of 0.6 Da and a detected protein threshold confidence of greater than 90%, and the UniProt Knowledgebase version 2007–0123 (or later), and set to detect contaminants (e.g. serum albumin) as described [29].

### Scratch Assay

HUVECs were plated in 6-well dishes in EBM-2 media and allowed to reach confluence (approximately 24 hr). A 200 μl pipette tip was used to scrape a “wound” in a vertical line. Cells were washed with PBS twice to remove unattached cells and incubated for 24 hr in EBM-2 (0.5% FCS and ½ the growth factor bullet kit) and BMP9. Pictures were taken 0 hr, 24 hr, and 48 hr (data not shown) after wounding using an Axiovert 40C microscope (Zeiss). Endothelial cell migration was quantitated by measuring the width of the cell-free zone (distance between the edges of the injured monolayer) as described previously [59].

### Mouse strains

Endoglin-targeted mice were screened for the presence of a neomycin resistance cassette in the truncated engineered endoglin allele as previously described [17]. Endoglin heterozygous (*eng* ^+/-^) mice were maintained in the C57BL/6 background. All experiments used male mice at 12–16 weeks of age. Breeding, maintenance, and experimentation were conducted according to the NIH standards established in the Guidelines for the Care and Use of Experimental Animals. All studies were approved by the Maine Medical Center Institutional Animal Care and Use and Institutional Biosafety Committees.

### Analysis of mouse tissues

Mice were weighed and euthanized at 12–16 weeks of age. All mice were genotyped twice: after birth and following sacrifice as described [20, 60]. Mice were anesthetized with avertin, and lungs were perfused with 8 ml of PBS at a constant flow rate using a butterfly needle in the right ventricle of the heart. Harvested lung tissue, single lobe, was fixed in 4% paraformaldehyde for 24 hrs and embedded in paraffin. Other lobes were divided and flash frozen (liquid nitrogen) for protein and mRNA analysis. Antibodies used for immunofluorescence and immunohistochemistry were: α-zyxin (Invitrogen), α-SMA-CY3 conjugated (Sigma), α-HU177 (Brooks Lab [61]), and α-CCL2 (Abcam). The slides were examined with a Zeiss Axioskop microscope (Thornwood, NY, USA). Imaging and color histogram analysis were performed using Image J software.

For HU177/MMP9 dual immunofluorescence, lungs from sacrificed mice were harvested and rinsed with PBS, transferred to OCT filled cryomolds and flash frozen on dry ice. Tissues were sectioned at 5μm and fixed in ice cold 50% methanol: 50% acetone solution for 1 min and allowed to air dry. Sections were blocked with 2.5% BSA in PBS for 1 hr at 37°C and washed with PBS. Sections were probed with HU177 (10ug/ml) and MMP9 (2μg/ml) in 2.5% BSA in PBS for 1 hr at 37°C and washed 3 times with PBS (5min each wash). Sections were incubated with 2° antibodies (1:2000) in 2.5% BSA in PBS for 1 hr at 37°C, and washed 3 times with PBS (5 min each wash). Sections were then incubated with DAPI (1:10000) 2.5% BSA in PBS for 5 min and washed with PBS. Fluoromount was placed on top of sections and sides were coverslipped and sealed with clear nail polish. Antibodies used were MMP9 (Abnova-PAB12714), Alexa Fluor 488 goat Anti-Mouse IgM- (Invitrogen Molecular Probes-A21042) and Alexa Fluor 594 goat Anti-Rabbit IgG- (Invitrogen Molecular Probes-A11012).

## Results

### Zyxin and endoglin localization to focal adhesions is BMP9 dependent

**Fig 1:**
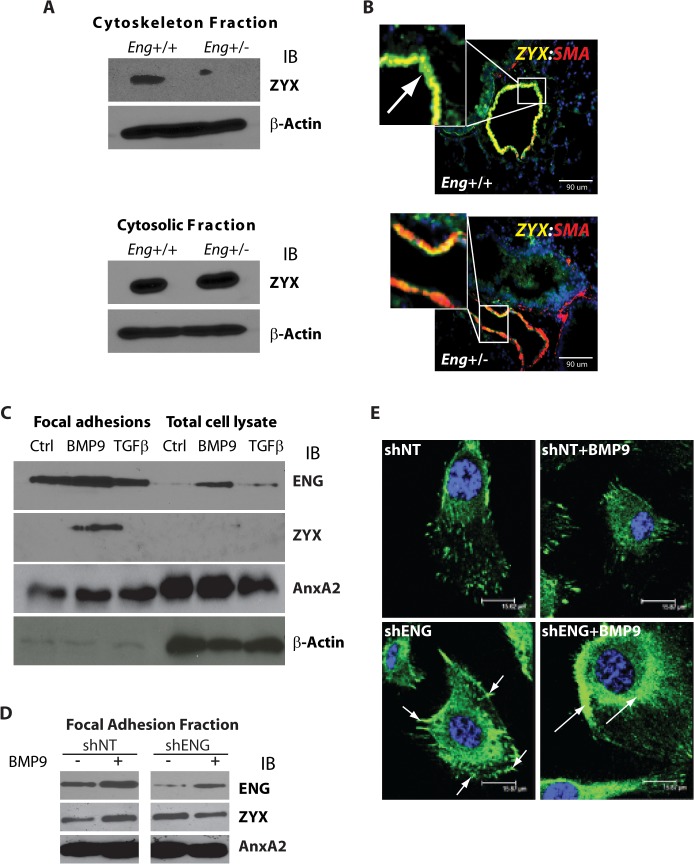
Zyxin localization in endothelial cells in response to endoglin and BMP9. (**A**) Western blot analysis of the QProteome-derived cytoskeletal fraction, including focal adhesion-associated proteins, and cytosolic fraction of wild type and *eng* ^+/-^ mouse lungs. (**B**) Double immunofluorescence confocal microscopy of wild type and *eng* ^+/-^ mouse lung tissue sections using α-zyxin and α-SMA antibodies. (**C**) Western blot of HUVEC focal adhesion and total cell lysate subcellular fractions (representative example of three replicates) following treatment with BMP9 or TGFβ (5ng/ml, Annexin A2; AnxA2). (**D**) Lentivirus-transduced control (shNT) and endoglin-targeting (shENG) short hairpin RNAs were used to suppress HUVEC endoglin expression; focal adhesion subcellular fractions were analyzed by Western blot. (**E**) Immunofluorescence analysis of zyxin localization using confocal microscopy. Anti-zyxin antibody and DAPI staining are in green and purple, respectively. Arrows indicate sites of focal adhesion (shENG) and the plasma membrane (shENG+BMP9), respectively. Scale bars indicate 15 μm.

HHT1 patients have an increased incidence of pulmonary AVMs [62, 63], though the development of AVMs remains poorly understood. To identify possible underlying signaling mechanisms, we examined the expression of zyxin in mouse lungs that are heterozygous for the targeted endoglin allele (*eng* ^+/-^) and littermate controls (eng+/+) [17]. Fractionation of the lung tissue into cytoskeletal and cytosolic fractions indicated that the *eng* ^+/-^ mice had lower levels of zyxin expression in the cytoskeletal fraction. Analysis of fractionated lung tissue from *eng* ^+/-^ mice showed decreased zyxin expression in the cytoskeleton fraction while expression in the cytosolic fraction was the same or slightly increased compared to wild type (Fig 1A). Though these are whole tissue fractions consisting of multiple cell types, they indicate a change in zyxin expression or localization. Zyxin expression has not been well characterized by histological analysis in normal lungs of mice or humans. However, based on GeoProfile data (NCBI), we anticipated its expression in multiple cell types throughout the lung. Looking at the *eng* ^+/+^ mice, we found zyxin to be differentially localized based on lung cell type. In epithelial cells lining the airways zyxin appeared to be localized in the cytosol. This observation contrasted with the cortical localization of zyxin in smooth muscle and endothelial cells, suggesting expression in both cell-cell junctions and cell-ECM junctions in the lungs (*eng* ^+/+^, Fig 1B, upper panels). Of greater significance, when we looked at zyxin staining in the *eng* ^+/-^ lungs we saw they exhibited decreased expression of zyxin as well as an altered localization staining pattern at the junction sites of smooth muscle and endothelial cells (Fig 1B, lower panels). Epithelial cell staining of zyxin remained unaffected in the *eng* ^+/-^ mouse lungs.

To further investigate the focal adhesion localization of endoglin and zyxin in endothelial cells, we stimulated HUVEC with SMAD1/5- and SMAD2/3-signaling ligands, BMP9 [24], and TGFβ1 [64, 65], respectively. Using an osmotic shock cell detachment method [58], we isolated a fibronectin bound focal adhesion fraction to analyze for protein composition following the mechanical removal of the adherent endothelial cells. While the focal adhesions isolated from the untreated and TGFβ1-treated HUVEC did not show detectable expression of zyxin, focal adhesions from the HUVEC treated with BMP9 showed a marked increase in zyxin expression (Fig 1C). This result suggests that zyxin expression in focal adhesions is independent of TGFβ signaling and is a Smad1/5-mediated response to BMP9 ligand stimulation and signaling. Endoglin was expressed in the focal adhesions of untreated, TGFβ1-treated, and BMP9-treated endothelial cells. However, western blotting of focal adhesion proteins from BMP9-treated HUVEC suggested increased endoglin expression when compared to control and TGFβ1-treated HUVEC (Fig 1C). Annexin A2, a membrane protein that is present in the cytosol, focal adhesion fraction and extracellular matrix fractions [66], provides both a normalization control between samples within each subfraction and, along with β-actin (and zyxin, [36]), an indication of enrichment for focal adhesion proteins. These results suggest that BMP9-dependent localization of zyxin in sites of focal adhesion formation in HUVEC is associated with increased endoglin levels, consistent with studies indicating that endoglin and zyxin are interacting proteins [43, 44]. Analysis of HUVEC total cell lysates (TCL) showed a decrease of both endoglin and zyxin when removed from tissue culture plate by trypsin suggesting that there is a relocalization of the proteins to focal adhesions, not a change in the total amount of the proteins (Fig 1C). qRT-PCR analysis of HUVEC mRNA indicated that these decreases are a post-transcriptional effect; only a modest upward trend in zyxin mRNA expression was observed upon BMP9 stimulation (data not shown). Thus, the relocalization of zyxin and endoglin to focal adhesions upon BMP9 stimulation is consistent with a less migratory [43], more quiescent [67] endothelial cell phenotype in response to BMP9 signaling.

To determine if zyxin localization was dependent on endoglin expression and localization, lentiviral shRNA was used to suppress endoglin expression in HUVEC [20, 29]. Using this approach, we have previously shown that the efficiency of endoglin knockdown in HUVEC approaches 50% in untreated, and about 70–80% in BMP9-treated cells [29]. BMP9 treatment of non-targeting control shNT-expressing HUVEC resulted in a similar coordinate increase in endoglin and zyxin levels in the focal adhesion fraction (Fig 1D). Focal adhesion fractions from HUVEC stimulated with BMP9 and expressing shNT or endoglin knockdown (shENG) indicated that when endoglin expression was reduced using shRNA in control BMP9-untreated HUVEC, there was an increase of zyxin expression in the focal adhesion fraction. However, there was a decrease of zyxin in the BMP9-treated shENG focal adhesion fraction, suggesting that reduced endoglin levels caused a loss of zyxin from the focal adhesion protein fraction (Fig 1D). Consistent with these results, immunofluorescent staining of HUVEC with anti-zyxin antibody showed that knockdown of endoglin expression led to an increase in protein staining and enhanced localization of zyxin in focal adhesions (Fig 1E, shNT, arrow versus shENG, arrows), which appeared to be independent of BMP9 treatment. Also consistent with immunoblot data (Fig 1D), immunofluorescence microscopy suggested a loss of zyxin from focal adhesions to the plasma membrane and cytoplasm (Fig 1E, shENG+BMP9, arrows) following BMP9 treatment. Though zyxin did not appear decreased in the shENG HUVECs as anticipated, these results suggest a role for BMP9 cooperation with endoglin in the localization of zyxin in endothelial cells, and support the idea that reduced zyxin in the cell-cell and ECM-cell junctions in the *eng* ^+/-^ mouse lungs potentially renders vessels more vulnerable to AVM formation.

### YAP1 as a SMAD- and endoglin-dependent effector of BMP9 signaling

**Fig 2:**
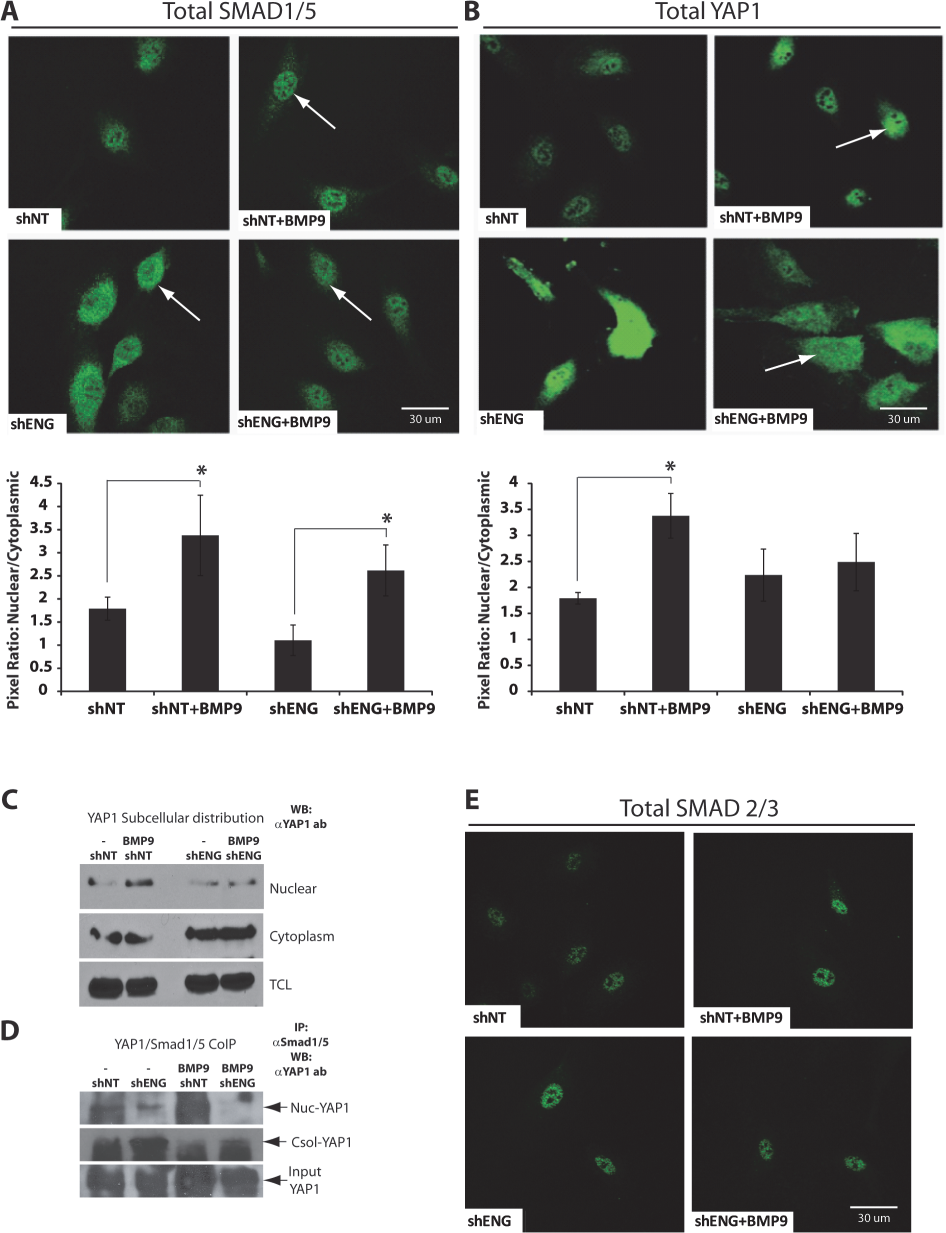
Endoglin- and BMP9-dependent localization of SMAD1/5 and YAP1. ShNT and shENG were used to suppress HUVEC endoglin expression. (**A-B, E**) immunofluorescence analysis using confocal microscopy shows that suppression of endoglin expression results in the loss of BMP9 dependent SMAD1/5 (**A**) and YAP1 (**B**) localization in the nucleus and increases their expression in the cytoplasm (arrows). The ratios of the immunofluorescence intensities measured in the cell nucleus versus cytoplasm are shown below panels A and B. The asterisk indicates a p value < 0.05. (**C**) Western blot analysis of YAP1 localization in HUVEC nuclear and cytoplasmic subcellular fractions, and total cell lysate (TCL). (**D**) SMAD1/5 and YAP1 coimmunoprecipitation show BMP9 dependent nuclear localization of the bound complex and cytoplasmic retention when endoglin is suppressed. (**E**) SMAD2/3 expression and localization appear unaffected in response to BMP9 and endoglin suppression.

Two endoglin signaling pathway members, zyxin and SMAD1/5, have been independently implicated in the regulation of the Hippo pathway [46, 68]. The Hippo pathway transcription coactivator, YAP1, has been shown to be downstream of zyxin [46] and to bind SMAD1/5 [69–71]. Therefore, we asked if there was potential for endoglin and BMP9 crosstalk with the Hippo pathway in endothelial cells. We first looked at HUVEC for changes in YAP1 and SMAD1/5 localization in response to BMP9 stimulation. Because SMAD1/5 and YAP1 have been shown to form a complex and alter Smad2/3 and Smad1/5 signaling [70], we looked to see if BMP9 treatment and/or endoglin knockdown caused localization changes of these proteins to the nucleus. As expected, BMP9 treatment of HUVEC increased the proportion of SMAD1/5 in the nucleus versus the cytoplasm (Fig 2A). Interestingly, HUVEC transduced with shENG showed an increase of SMAD1/5 in the cytosol in BMP9-untreated cells, suggesting impairment of basal signaling that is endoglin- and SMAD1/5-dependent. This cytosolic relocalization of SMAD1/5 was partially rescued by BMP9 treatment, suggesting a role for endoglin, a BMP9 target as well as BMP9 signal transducer [29], in the nuclear localization of SMAD1/5 in response to BMP9 signaling. Next, we examined HUVEC for the localization of YAP1 in response to BMP9 treatment. We found translocation of YAP1 from the cytoplasm to the nucleus (Fig 2B, upper panels; shNT versus shNT + BMP9). YAP1 and SMAD1/5 had similar localization patterns and BMP9 appeared to promote both their nuclear localization in HUVEC. Following endoglin knockdown, YAP1 remained localized in the cytosol, similar to that seen for SMAD1/5 at the basal level (Fig 2B lower panels). Finally, BMP9 showed diminished capacity to rescue the nuclear localization of YAP1 as seen for SMAD1/5 (the relative pixel densities for nuclear versus cytoplasmic staining are indicated below Fig 2A and 2B). This result supports the hypothesis that endoglin promotes BMP9-dependent translocation of YAP1 to the nucleus.

Next, we performed cell fractionation followed by Western blot analysis of HUVECs after BMP9 stimulation and/or shENG transduction. These observations confirmed the immunofluorescence results indicating that BMP9 increased the nuclear localization of YAP1, and that this effect was impaired by shENG knockdown (Fig 2C). YAP1 expression was also examined at the mRNA level by qRT-PCR. There were no significant changes elicited by BMP9 treatment or knockdown of endoglin expression (data not shown), suggesting YAP1 localization, but not expression, are BMP9 and endoglin responsive. Nuclear coimmunoprecipitation using anti-SMAD1/5 antibody indicated that BMP9 potently induced a YAP1-SMAD1/5 complex in the nucleus. This complex was nearly eliminated by endoglin shRNA knockdown (Fig 2D). BMP9 treatment did not significantly alter levels of the YAP1-interacting protein, 14-3-3 ζ, as determined by Western blot ([72], data not shown), indicating that 14-3-3 ζ expression remains unaltered in BMP9-dependent YAP1 localization or degradation. These data suggest that BMP9 and endoglin regulate nuclear localization of the Hippo pathway transcription coativator, YAP1, via its binding to SMAD1/5, and support a mechanism whereby BMP9 signaling modulates YAP1 activity in an endoglin-dependent manner at the levels of transcription factor/cofactor interaction.

We examined SMAD2/3 localization in our *in vitro* endothelial cell system because SMAD2/3 and SMAD1/5 have both been shown to directly interact with the Hippo pathway transcription coactivator, YAP1 [70]. Consistent with data shown in Fig 1 indicating no effect of TGFβ1 signaling, immunofluorescence confocal microscopy of SMAD 2/3 showed little to no change in its expression pattern in response to BMP9 (Fig 2E). While there was no change in expression, the staining of SMAD 2/3 remained mostly nuclear suggesting basal TGFβ signaling, likely in response to the fibronectin-coated slides. These results support the hypothesis that in endothelial cells, Hippo crosstalk via YAP1 is regulated by BMP9 signaling via endoglin.

### CTGF/CYR61 are endoglin-dependent effectors of BMP9 signaling in endothelial cells

To further identify the target gene transcription regulation of the YAP1/SMAD1/5 complex in the nucleus, we examined the YAP1 target genes CTGF and CYR61. Because CTGF and CYR61 are regulated by BMP signaling in other cellular contexts [53, 54], we sought to clarify the consequences of BMP9 signaling in terms of the role of endoglin, SMAD1/5, and YAP1 in the mechanism of BMP9-dependent CTGF and CYR61 expression.

**Fig 3:**
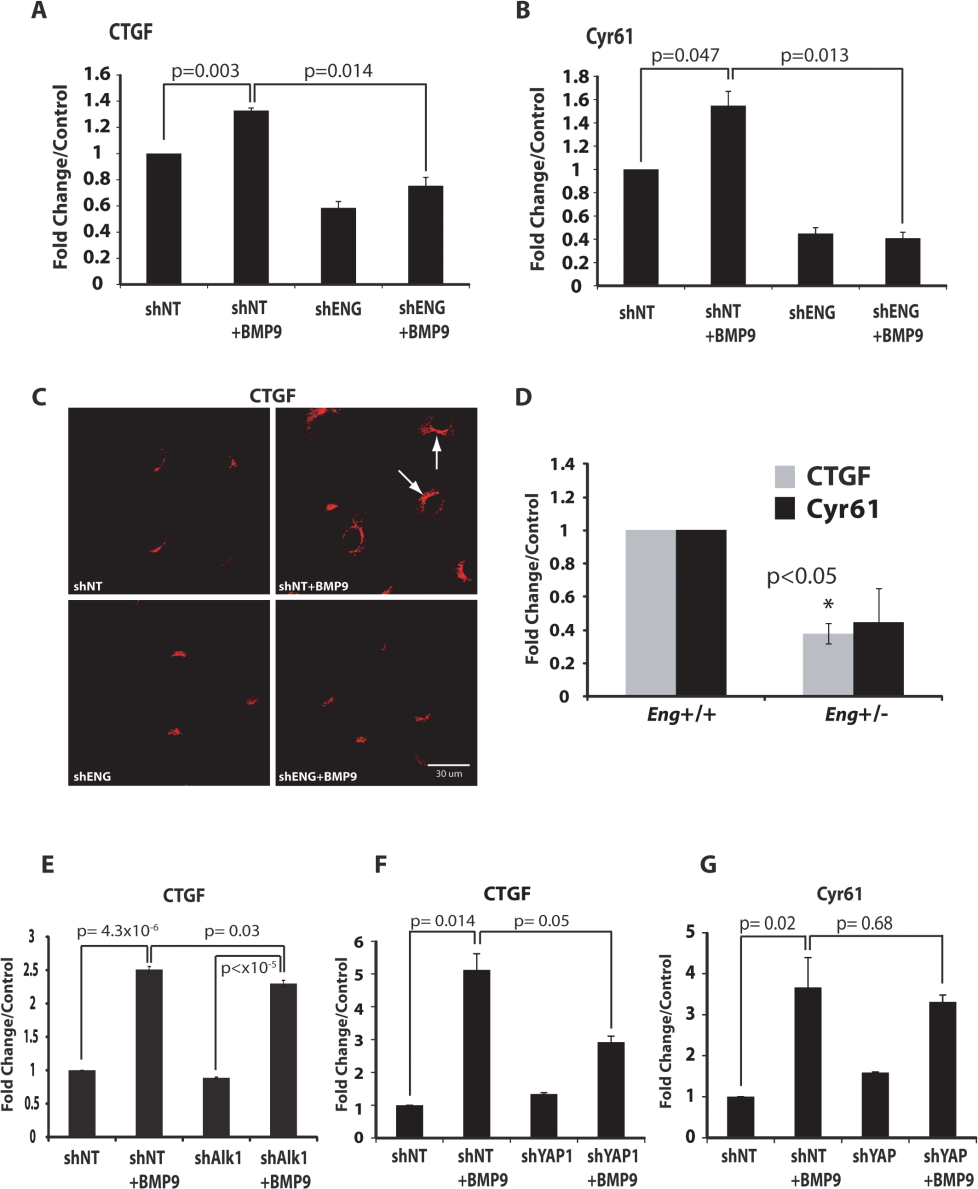
YAP1 targets CYR61 and connective tissue growth factor (CTGF) expression and secretion are regulated by BMP9 and endoglin. All cells were plated on fibronectin. (**A-B**) Quantitative real-time RT-PCR (qRT-PCR) of (**A**) CTGF and (**B**) CYR61 following BMP9 treatment and endoglin suppression using shRNA. (**C**) Immunofluorescence confocal microscopy analysis of CTGF in response to BMP9 treatment and endoglin suppression. (**D**) qRT-PCR of CTGF (gray) and CYR61 (black) mRNA from wild type and *eng* ^+/-^ mouse lung mRNA preparations. (**E**) qRT-PCR of CTGF following BMP9 treatment and Alk1 suppression using shRNA. (**F-G**) qRT-PCR of (**F**) CTGF and (**G**) CYR61 following BMP9 treatment and YAP1 suppression using shRNA. Human cyclophilin primers were used for normalization.

First, we determined whether BMP9 and endoglin regulate expression of YAP1 target genes, CTGF and CYR61, in HUVEC. Cells were plated on fibronectin to provide an ECM environment that resembles a Hippo-responsive context [73] but, unlike more rigid matrices, does not independently activate YAP1 signaling [74]. Under these conditions, CTGF mRNA expression was upregulated in BMP9-stimulated HUVEC as determined by qRT-PCR (Fig 3A). A similar result was obtained for the expression of CYR61 (Fig 3B). Importantly, shRNA knockdown of endoglin potently repressed both CTGF and CYR61 expression in response to BMP9 (Fig 3A and 3B), indicating a requirement for endoglin in the induction of these proteins by BMP9 in endothelial cells. Though anti-CYR61 antibodies proved problematic, increased cellular protein expression of CTGF in BMP9-treated HUVEC was confirmed by immunofluorescence confocal microscopy (Fig 3C). Consistent with our data demonstrating that when endoglin expression was suppressed, expression of CTGF was impaired (Fig 3A), *in vivo* data revealed that CTGF expression was significantly reduced in *eng* ^+/-^ mouse lung mRNA preparations analyzed by qRT-PCR (Fig 3D). Similar to CTGF, CYR61 expression trended lower in *eng* ^+/-^ lung tissue, though this did not reach statistical significance (p = 0.08, Fig 3D).

To further investigate the specificity of the BMP9-dependence of CCN expression in endothelial cells, we used shRNA to knockdown the Alk1 receptor. The response of CTGF to BMP9 was significantly though partially repressed by Alk1 knockdown (Fig 3E). We did not see a significant effect for Cyr61 (data not shown). This latter result may be due to expression of endogenous BMPs giving a higher baseline for Cyr61 expression. Finally, we tested the effect of YAP1 shRNA lentiviral knockdown in the response of CTGF and CYR61 to BMP9 treatment. YAP1 knockdown slightly elevated basal CTGF and CYR61 levels as determined by qRT-PCR (Fig 3F and 3G). Despite the elevated basal level of CTGF, YAP1 knockdown strongly repressed CTGF induction by BMP9 (Fig 3F). As above, a similar trend was observed for CYR61, though this response also did not achieve statistical significance (Fig 3G), again, potentially reflecting elevation of basal CYR61 relative to CTGF. Together, these data support the hypothesis of crosstalk between BMP9 and Hippo pathways involving cooperative BMP9, endoglin, and YAP1 interactions that regulate CTGF and CYR61 expression.

**Fig 4:**
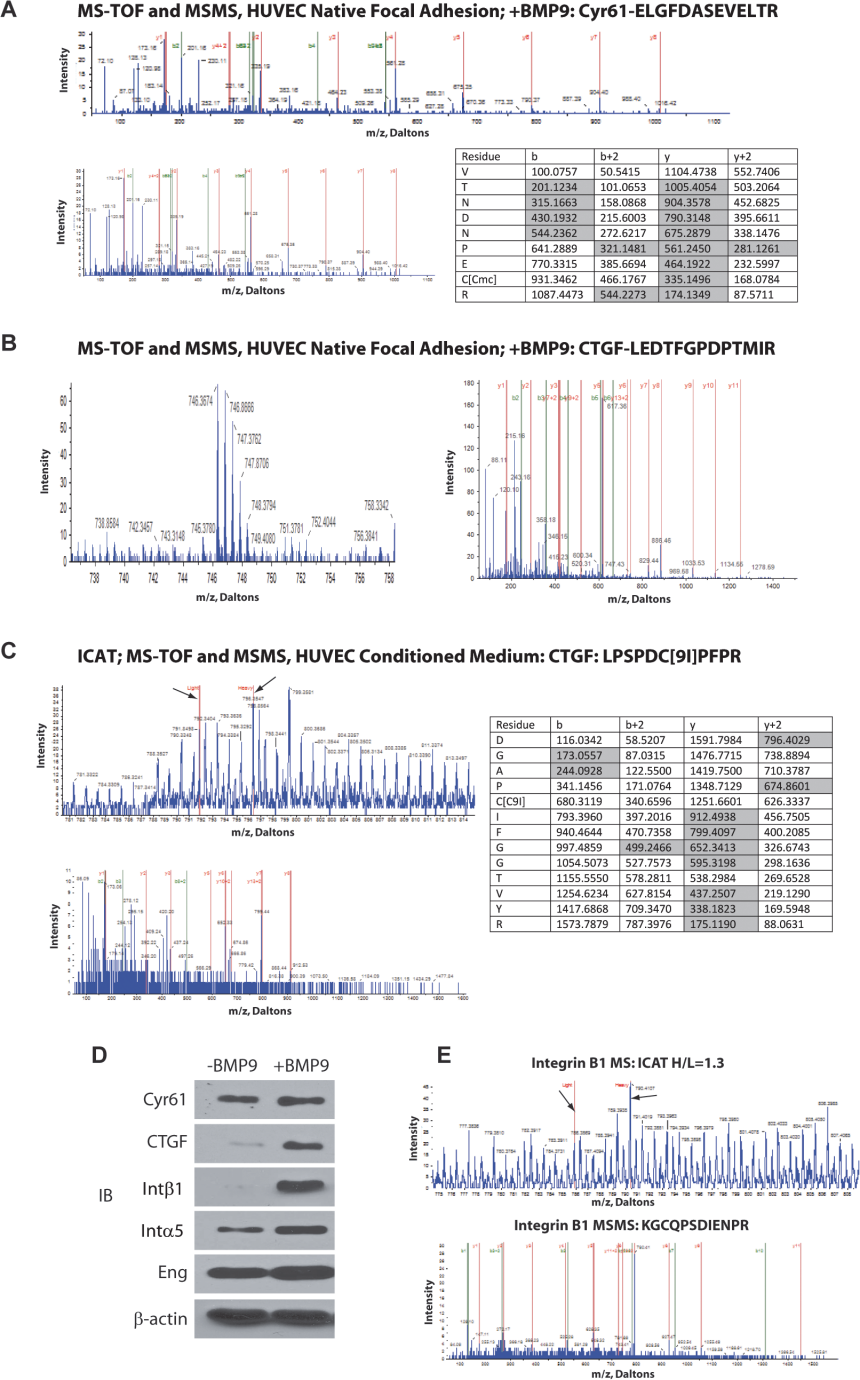
Mass spectrometric analysis suggests BMP9-dependent CTGF and CYR61 secretion and focal adhesion localization. **(A-B)** Survey spectra of BMP9-treated HUVEC focal adhesion preparations indicate the presence of CYR61 (**A**) and CTGF (**B**) proteins in focal adhesion fractions. MS identification and MSMS collision-induced decay sequence spectra are shown in the top and bottom spectra panels, respectively. Sequence data are presented in the table showing expected and observed ions (shaded) for this protein. (**C)** Isotope-coded affinity tag (ICAT) mass spectrometry of control and BMP9-treated and control HUVEC conditioned medium samples. Heavy isotope BMP9-treated and light isotope-tagged control peptide spectra are indicated with h or l, respectively. Left panels (**A-C**) are MS-TOF analyses for identification (**A-C**) and quantitation (**C**). (**C**) Left panel shows an example of heavy- (BMP9-treated) and light-isotope-tagged (control) CTGF peptide ICAT-labeled ions (arrows indicate ICAT-ions); right panels are MSMS-TOF collision-induced decay-based protein sequence analyses, used to identify the indicated peptides for CTGF. (**D**) Western blot analysis of HUVEC focal adhesion subcellular fraction for CYR61, CTGF, and integrins β1 and α5 proteins with or without BMP9 treatment. (**E**) Mass spectrometry ICAT analysis of the HUVEC focal adhesion subcellular fraction for integrin β1.

Based on the established functions of CTGF and CYR61 in matrix metabolism, vessel maturation, [75, 76] and Hippo/YAP1 signaling [50], we hypothesized a BMP9- and endoglin-dependent function in which endothelial cell expression and secretion of CCN1/2 matricellular proteins is a YAP1/SMAD1/5-dependent response that alters ECM metabolism and the inflammatory response during angiogenesis. To examine whether CCN1 and CCN2 matricellular proteins were in fact being secreted and engaging the ECM proteins at endothelial cell sites of adhesion, we used mass spectrometry-based protein analysis of focal adhesion fractions [58]. HUVEC focal adhesion fraction protein identification and ICAT-based quantitation [29] suggested that CTGF and CYR61 are constituents of the endothelial cell/ECM contacts when the cells were stimulated with BMP9 (Fig 4A and 4B). Unstimulated control focal adhesion fractions from HUVEC did not yield CCN1/2-characteristic ions (data not shown). We also examined the conditioned media of BMP9-treated HUVEC compared to untreated HUVEC using ICAT-based mass spectrometry [77] as previously described [29]. These results indicated that there is an increase of CTGF secreted into the conditioned medium in response to BMP9 (Fig 4C, arrows). Identified proteins showing altered expression levels in conditioned media in response to BMP9, including CTGF, suggest a broader endothelial cell response (Table 1). Consistent with the foregoing results, Western blot analysis confirmed the presence and increased levels of CTGF and CYR61 in the focal adhesion fraction in response to BMP9 stimulation (Fig 4D). These data suggest a mechanism by which endothelial cell-autonomous regulation and secretion of matricellular proteins, in response to BMP9 and endoglin, may contribute to altered ECM structure, function, and cell-ECM interactions.

**Table 1:** Proteins identified as up-regulated and down-regulated in CM from HUVEC +/- BMP9 ICAT data.

No.	Acc #	Protein Name	Regulation	H:L
1	O75916	Regulator of g-protein signaling 9	up	>5
2	Q9NSI6	Bromodomain and WD repeat-containing protein 1	up	3.92
3	Q6FHL8	CTGF protein	up	2.16
4	P13987	CD59 glycoprotein	up	1.78
5	P09382	Galectin-1	up	1.40
6	Q9P283	Isoform 2 of Semaphorin-5B	up	1.13
7	Q60FE2	MYH9 variant protein	down	0.76
8	Q5W0X3	FK506 binding protein 1A	down	0.65
9	P00734	Prothrombin	down	0.60
10	B4E351	Insulin-like growth factor-binding protein 4 (IGFBP4)	down	0.50
11	A0AV88	ADAM10 protein	down	0.41
12	F2Z2G2	Elongation factor 1-beta	down	0.20

To further investigate this idea we sought to determine potential functional downstream consequences of CYR61 and CTGF expression. CTGF mediates TGFβ-dependent fibronectin synthesis [78] Fibronectin is a matrix ligand for integrin α5β1 and CYR61 is known to regulate β1 integrin [79]. Since integrin β1 has been implicated in endoglin signaling and protein-protein binding capacities, we hypothesized that if BMP9 functionally induces CYR61/CTGF expression, then localization of integrins α5 and β1 would be altered in focal adhesions. Indeed, as suggested by mass spectrometry (Fig 4E, arrows) and western blotting, focal adhesion enrichment of integrin β1 and α5 was observed in response to BMP9 stimulation (Fig 4D). This result is supportive of an important role for integrin-rich focal adhesion composition via BMP9 regulation of CTGF/CYR61 expression.

### BMP9 and endoglin in the metabolism and remodeling of the ECM

**Fig 5:**
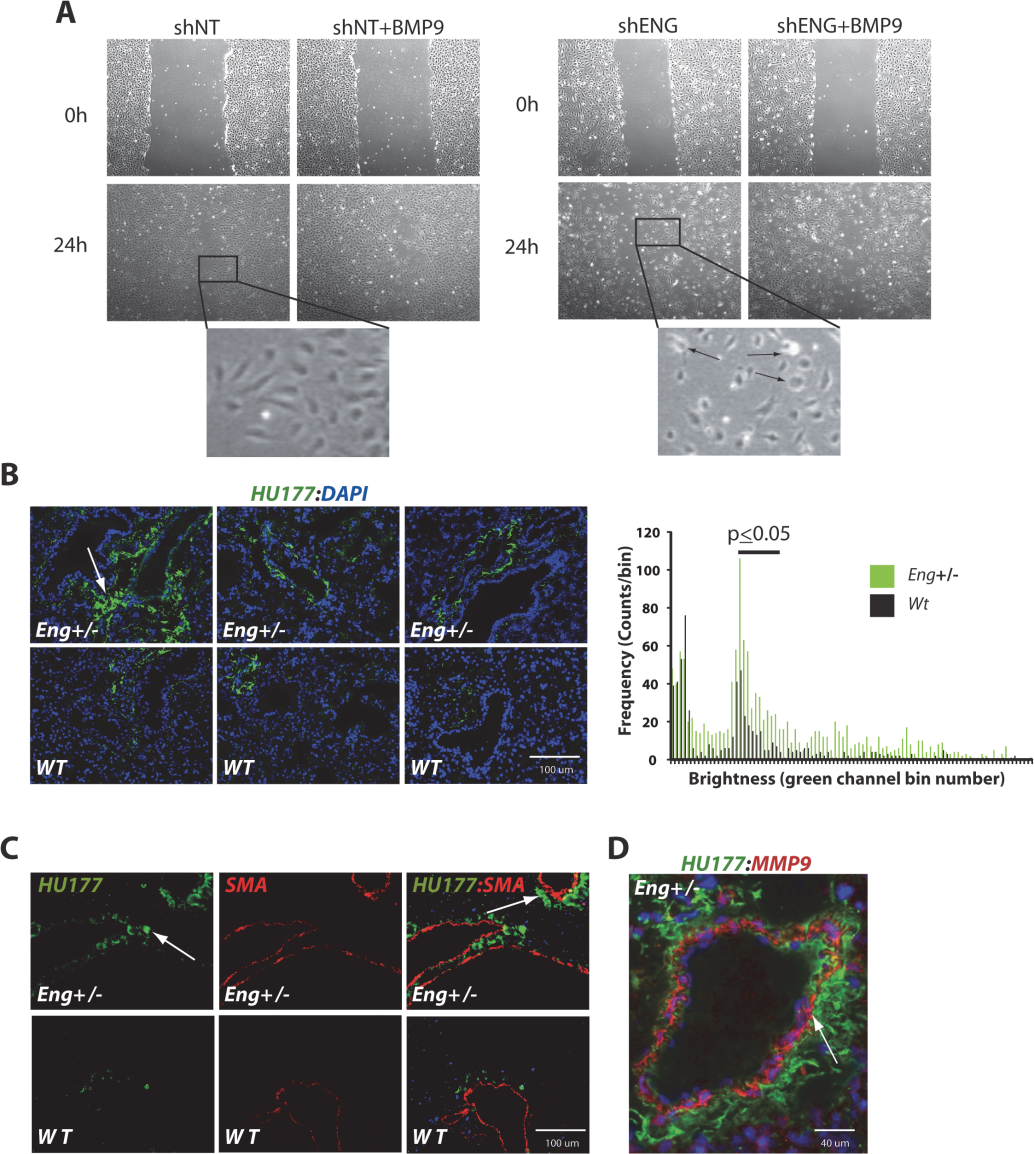
Altered wound healing patterns in endoglin-deficient HUVEC and extracellular matrix (ECM) remodeling in *eng* ^+/-^ mouse lungs. (**A)** In vitro scratch assay with HUVEC and endoglin deficiency obtained at 24 and 48 hrs after “wounding”. Insets exemplify altered morphology seen with endoglin knockdown (24 hr shENG, arrows). (**B**) Immunofluorescent visualization of collagen exposure using the collagen cryptic epitope-specific antibody, HU177 [81]. Accompanying ImageJ color histograms show total pixel counts for the green HU177 channel. Horizontal bar indicates value differences with p<0.05. (**C**) Dual immunofluorescence microscopy obtained from wild type and *eng* ^+/-^ mouse lungs using HU177 and smooth muscle alpha actin (SMA) antibodies. Arrows indicate regions of exposed collagen cryptic epitope (α-HU177), shown predominately in the *eng* ^+/-^ mouse lung. (**D**) Dual immunofluorescence analysis of *eng* ^+/-^ mouse lung sections for HU177 (green) and MMP9 (red) Arrow exemplifies adjacent staining).

Identifying CTGF and CYR61 as direct target genes of BMP9- and endoglin-dependent signaling via the binding and nuclear translocation of the transcription complex, YAP/SMAD1/5, suggests they participate in important mechanotransductive and angiogenic events involving focal adhesion formation and ECM regulation. We conducted a “wound healing” or “scratch” assay [80] to identify how the malformed focal adhesions, ECM turnover, and improper angiogenic signals were affecting these signaling mechanisms. Again using shNT and shENG lentivirus-transduced HUVEC, stimulated or not with BMP9, we mechanically formed a wound and looked at two time points for wound closure. At 24 hr, cells had migrated into the wound area of both the shNT and shENG independent of BMP9 (Fig 5A). As expected, the shNT HUVEC migrated into the wound with leading cells followed by a sheet like formation, common to endothelial cells in these experiments, as the wound closed at 48 hr. (data not shown). Interestingly, although the shENG cells migrated into the wound area at 24 hr and closed the wound by 48 hr (data not shown), they did not maintain the sheet like formation that is normally seen in endothelial cell wound healing assays [59]. Specifically, the shENG HUVEC exhibited disoriented migration, cluster formation, and appeared rounded up and under light microscopy (Fig 5A, arrows in shENG, inset). These results suggest that a deficiency in endoglin leads to improper endothelial cell migration in response to wounding of endothelial cells that may reflect improper focal adhesion formation and/or cell-ECM contact.

To assess the potential for endoglin deficiency alterations in the ECM structure *in vivo*, we examined *eng* ^+/-^ and wild type adult mouse lungs for changes in the conformation or turnover of type I and type IV collagen, constituents of the ECM in the lungs. The HU177 antibody detects a specific cryptic epitope exposed by collagen I and collagen IV as a result of matrix turnover or degradation, which occurs during neovascularization [81] and vascular reperfusion [61]. Therefore, we looked at immunofluorescence staining of wild type *eng* ^+/+^ and mutated *eng* ^+/-^ mouse lung sections with HU177 antibody. Immunofluorescence analysis showed increased HU177 cryptic epitope exposure in the ECM (Fig 5B), which was adjacent to the alpha smooth muscle actin (SMA) positive smooth muscle cells of the arteries, stained with smooth muscle actin (SMA) (Fig 5C). These results are consistent with enhanced degradation of the ECM in endoglin haploinsufficiency and provide a novel functional probe of vessels with altered remodeling due to reduced endoglin expression. Cryptic epitope exposure is functionally generated by the degradation of the ECM by matrix metalloproteinases, including MMP9, and we previously showed that MMP9-null mice exhibit reduced cryptic epitope exposure [61]. Consistent with these studies and the preceding results, dual immunofluorescence analysis revealed MMP9 expression in *eng*+/- lung vessels adjacent to HU177+ antibody reactivity (Fig 5D). As above, there was much less HU177 staining in wild type vessels, consistent with Fig 5B and 5C (data not shown). These data suggest that the vessel ECM and microenvironment in *eng*+/- mice is less quiescent and more angiogenic.

### BMP9 versus CCN1/2 regulation of endothelial chemokine axes: CCL2/CCR2

**Fig 6:**
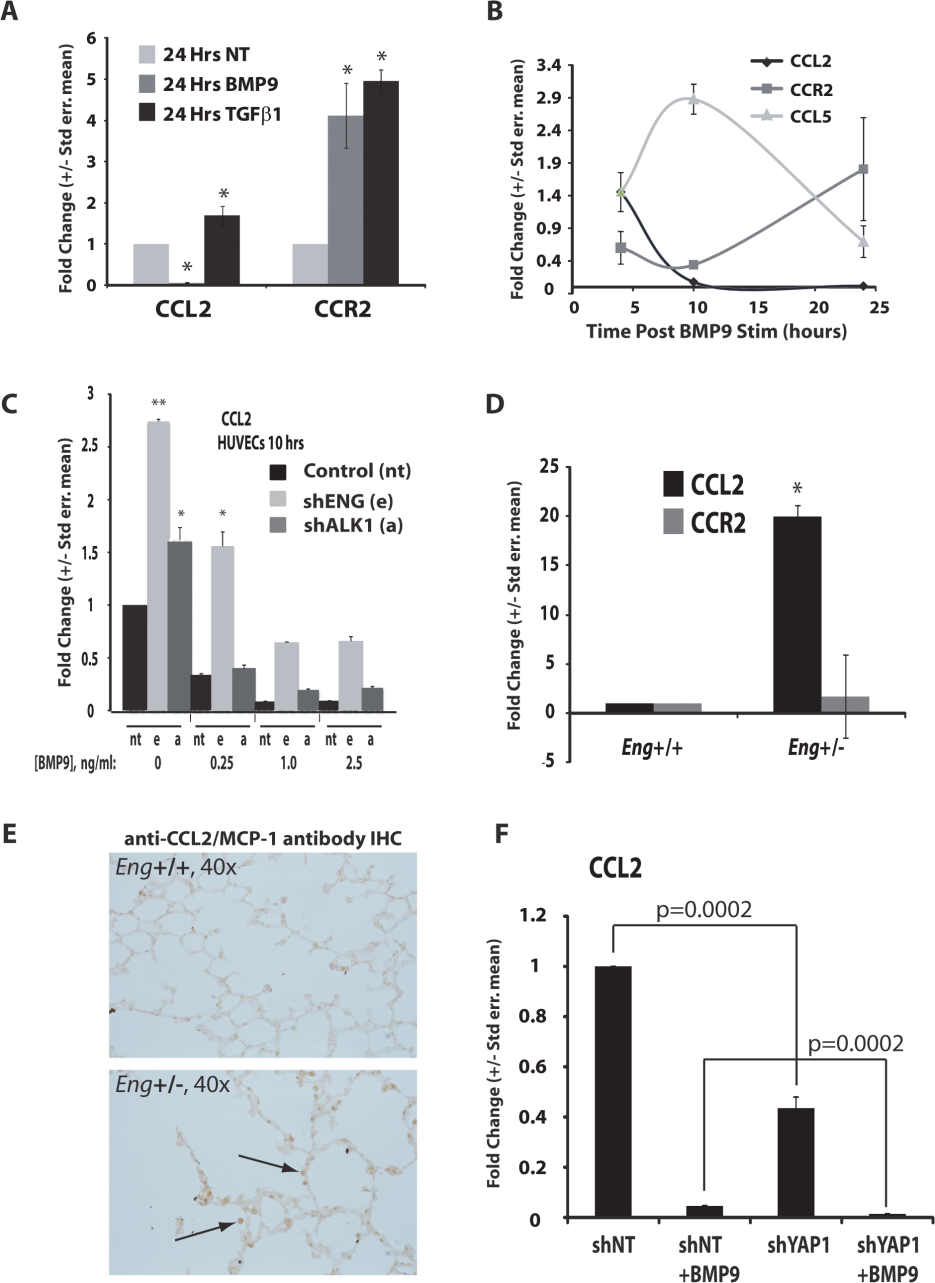
Inflammatory cytokines are regulated by BMP9 and endoglin expression and signaling. **(A)** CCL2 and CCR2 levels of expression in HUVEC following treatment with TGFβ or BMP9, as compared to control (NT). **(B)** qRT-PCR analysis of time course of BMP9-dependent changes in mRNA levels for CCL2, CCR2, and CCL5. **(C)** Relative to control lentivirus (NT), qRT-PCR indicates shRNA for endoglin or ALK1 (e, a, respectively) rescues BMP9-dependent repression of CCL2 expression. **(D)** CCL2 and CCR2 expression in WT (n = 3) and *eng* ^+/-^ (n = 3) mouse lung mRNA preparations. *, p<0.025; **, p<0.001. Human and mouse β2-microglobulin was used for normalization. **(E)** Monocyte infiltration and α-CCL2 expression in *eng* ^+/-^ lungs, as compared to wild type, *eng* ^+/+^. **(F)** qRT-PCR analysis of BMP9-dependent CCL2 expression with YAP1 knockdown.

**Fig 7:**
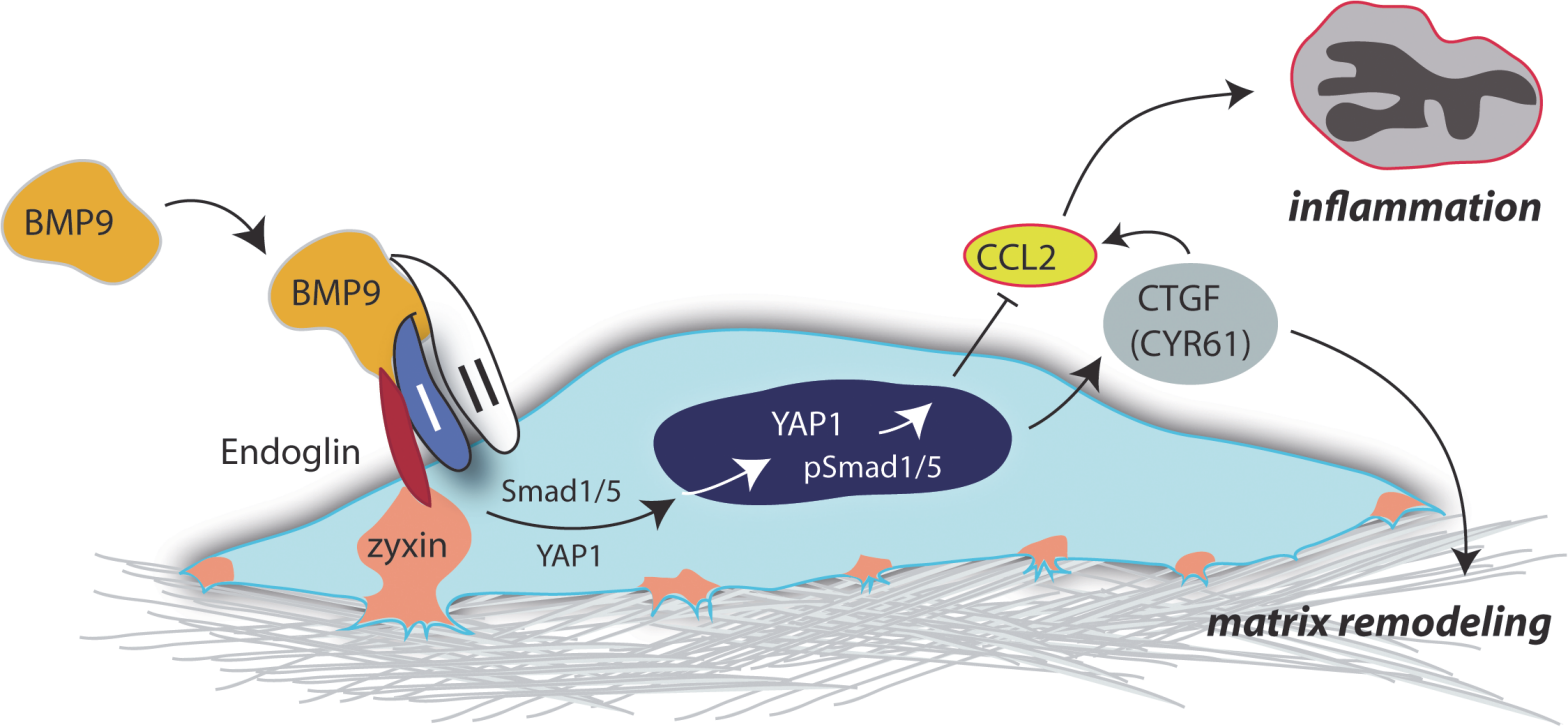
Integration of BMP and Hippo signaling in endothelial cells. We hypothesize that BMP9 regulates the expression and secretion of matricellular proteins, CYR61 and CTGF, via crosstalk between the BMP (endoglin, ALK1, and SMAD1/5) and Hippo (Zyxin, YAP1, SMAD) signaling pathways. We suggest that the secretion of CYR61/CTGF regulates the composition and function of focal adhesion proteins, cell migration and ECM remodeling, and acts as a key effector of BMP9 signaling via the HHT target genes, ALK1 and endoglin.

The increased exposure of the cryptic epitopes of collagen suggests a localized pro-angiogenic state of the arteries in the *eng* ^+/-^ mouse lungs, as compared to their littermate controls. Moreover, the identification of infiltrating inflammatory cells by MMP9 expression suggests increased inflammation in the lungs of the *eng* ^+/-^ mice as well. CTGF may regulate proinflammatory responses including monocyte recruitment [56]. Because CYR61 [82] and CTGF [56, 83] can promote the expression of monocyte chemoattractant protein 1 (MCP-1/CCL2), we examined the CCL2/CCR2 pro-inflammatory axis in response to BMP9 signaling. Our data confirmed that CCL2, a TGFβ1 target gene and inducer of angiogenesis [84], was upregulated, as expected, by TGFβ1 treatment (Fig 6A). In striking contrast, CCL2 was profoundly repressed by BMP9 treatment, while its cognate receptor, CCR2, was upregulated by both BMP9 and TGFβ1 treatment (Fig 6A). Repression of CCL2 was rapid and essentially complete 10 hr after treatment (Fig 6B). The idea that BMP9 differentially regulates the endothelial cell inflammatory chemokine signature [29] is further supported by the observation that CCL5 expression is transiently upregulated and then decreased following BMP9 treatment, in opposition to CCL2. The expression of the CCL2 receptor, CCR2, steadily increases during this time course (Fig 6B). Lentiviral shRNA studies confirmed that the BMP9-dependent repression of CCL2 was mediated by both endoglin and ALK1 (Fig 6C), supporting the view that these proteins cooperate to regulate CCL2 repression by BMP9 in endothelial cells. *In vivo* analysis by qRT-PCR of the *eng* ^+/-^ mouse lung also showed increased CCL2 mRNA expression, suggesting reduced responsiveness to BMP9-mediated repression of CCL2 and the potential for an increased inflammatory response when compared to wild-type levels of endoglin (Fig 6D). Histological analysis of CCL2 expression in the *eng* ^+/-^ mouse lung confirmed that, *in vivo*, CCL2 expression increased in both the vasculature and infiltrating inflammatory cells (Fig 6E). Finally, knockdown of YAP1 additively repressed basal and BMP9-treated CCL2 expression (Fig 6F, second and fourth bars). These results provide new insight that BMP9 may independently regulate the CCN1/2 and CCL2/CCR2 axes by distinct pathways (e.g., repression of CCL2 by BMP9 is dominant and distinct from CCL2 induction by CTGF/CYR61) and that Hippo/YAP1 signaling may promote CCL2 expression using a pathway that is distinct from BMP9/ALK1 (Fig 7).

## Discussion

Angiogenesis is the development of new blood vessels from existing ones. This is a multiple step process that involves the intricate orchestration of signaling events within endothelial cells to become active, change into tip and stalk cells, migrate, and return to quiescent endothelial cells in the new blood vessel. These events and their timing are mediated by endothelial cells and the stimuli they receive from growth factors, inflammatory molecules, cytokines, and the remodeling ECM. Endoglin is a key mediator of BMP9-dependent angiogenic signaling [29, 85, 86], though the understanding of its role in this process is still incomplete. Here we sought to explore, in more detail, how endoglin haploinsufficiency in endothelial cells may contribute to the abnormal angiogenic signaling that guides AVM formation. Our data suggest that one such mechanism involves a unique crosstalk between the BMP and Hippo pathways that leads to the regulation of secreted matricellular proteins and chemokines, cell migration and morphology, and ECM remodeling.

This study explores the interaction of endoglin and zyxin, and highlights the importance of signaling events that may result from their binding and localization. Our previous work identified zyxin as an endoglin binding protein and how it affected endoglin’s phosphorylation state and canonical TGFβ signaling [43]. We sought to further investigate the importance of this interaction by providing insight into the molecular mechanisms downstream of this interaction and to clarify how TGFβ1/BMP9 stimulation may be involved. Zyxin, a prominent player in cell-ECM mechanotransduction at focal adhesions [31], is important in integrin clustering and signaling events that control cell migration and morphology [87]. Here we identify BMP9, and not TGFβ1, as the ligand responsible for the colocalization of endoglin and zyxin in the focal adhesions of HUVEC. Interestingly, knockdown of endoglin caused a localization of zyxin to focal adhesions independent of BMP9 stimulation, but the affected endothelial cells became larger in size and their morphology became altered compared to the normal cobblestone morphology of control cells, shNT HUVEC. These changes in the morphology of endoglin-deficient endothelial cells have been shown by others [88–90], but our results suggest that an important function for endoglin is to fine-tune BMP9 signaling in its ability to regulate adhesive (zyxin) chemokines (e.g., SDF1, CXCL10 [29], and CCL2) and matricellular proteins (CTGF, CYR61), key regulatory determinants of cell adhesion and constituents of the ECM.

Analysis of the distal vasculature in the adult mouse lung, where endoglin expression is elevated [91]) showed zyxin expression to be located in the cell-cell and cell-ECM contact sites around the endothelial cells and smooth muscle cells of the arteries. Mouse lungs heterozygous for endoglin showed a decrease in this localization of zyxin and an increase in its cytosolic expression, further underscoring endoglin’s role in zyxin localization and suggesting its importance to the HHT vascular disease phenotype. Zyxin is a marker of mature focal adhesions *in vitro* [92], suggesting a role for this protein toward a more quiescent and less dynamic state of the vasculature *in vivo*. Therefore, we suggest that endoglin-deficient *eng* ^+/-^ mouse lungs exhibit impaired zyxin expression in the cell-cell and the cell-ECM contacts, thus contributing to a less quiescent, more angiogenic state. These results offer interesting new insight into the cell-ECM connection in the dynamic context of a tissue. The endothelial cell results presented here show that differential localization of zyxin between sites of focal adhesion and the cytosol and membrane compartments is effected by BMP9 treatment and endoglin levels. The precise control of zyxin localization may vary *in vivo* and *in vitro* due to differences in cell-cell and cell-ECM contact of the endothelial cells. These contacts may engage regulatory mechanisms *in vivo* that are not recapitulated in cell culture. However, because total zyxin protein levels are largely unchanged *in vivo* and *in vitro*, while its localization is effected by endoglin expression in both contexts, we suggest an important role for endoglin and zyxin in the mechanotransduction signaling events that take place in response to zyxin localization to focal adhesions.

To further address the downstream consequences of the endoglin/zyxin interaction taking place in the focal adhesions of endothelial cells in response to BMP9, we examined elements of the Hippo pathway. The Hippo pathway is important in potentiating cell proliferation, growth, differentiation, and apoptosis [68]. Its ability to coordinate these cellular processes suggests a key role in development, wound healing, and, now, endoglin- and BMP9-dependent effects on angiogenesis. The Hippo pathway transcription factor YAP1 has been strongly implicated as key player in cytoskeleton mediated signaling and ECM responsiveness [73, 93–95]. Other studies have implicated the SMADs and zyxin as regulators of the Hippo pathway, further supporting our finding that BMP9 and endoglin may act as key components in the localization of YAP1 via its binding to SMAD1/5. However, the signaling mechanisms are still poorly understood and key components of this pathway are still being identified.

Zyxin was identified [46] as a component of the Hippo pathway using a genetic screen in Drosophila. In this model, a decrease in zyxin resulted in the depletion of nuclear Yorkie (YAP1). In this study, endoglin knockdown caused zyxin to be localized in focal adhesions and YAP1/SMAD1/5 to be sequestered to the cytosol of endothelial cells, suggesting a mechanism by which YAP1 and zyxin are coordinately regulated. We were able to show that CTGF induction by BMP9 appears to be YAP1-dependent, thus providing the additional insight that BMP9 signaling may interact with the Hippo pathway. As with *in vivo* reduction of CTGF and CYR61 levels in endoglin haploinsufficient mouse model of HHT (Fig 3D), CTGF repression was more pronounced than CYR61. The YAP1 knockdown experiments suggested that basal CTGF and CYR61 levels were enhanced, whereas BMP9-dependent induction of these proteins required YAP1. These data suggest that future studies of the role of CCN proteins in HHT can focus on potential matrix-dependent YAP1-inhibitory,-promoting, and-independent pathways regulating CTGF and CYR61 expression. Potential YAP1 inhibitory signals could include protein degradation. However, BMP9 treatment did not alter cytoplasmic 14-3-3 ζ levels, regardless of endoglin knockdown (data not shown), thereby excluding this potential mechanism contributing to altered cytosolic sequestration and degradation of YAP1. Though BMP9 treatment was able to partially rescue SMAD1/5 nuclear localization, most likely in response to ALK1 continuing signal transduction, zyxin and YAP1 remained mislocalized. This observation suggests an endoglin-dependent mechanism by which cytoskeletal-mediated responses are disrupted and downstream signaling is misregulated. This study thus elucidates a potential role for BMP9 and endoglin in the Hippo pathway, and we hypothesize that its regulation is important for mechanotransduction and angiogenesis.

It has been shown that in vascular injury models, including ischemia, that CTGF expression is induced in endothelial cells and that endothelial cells are one of the main sources of CTGF secretion in vascularized tissue [96, 97]. This secreted matricellular protein’s ability to regulate adhesive signaling and to bind to the ECM protein fibronectin, focal adhesion-associated receptors, and integrins suggests a role in regulating ECM function in the angiogenic process. CTGF has also been shown to influence growth factor signaling, including promoting TGFβ as well as inhibiting VEGFA and BMP4. The regulation of CTGF expression and secretion by BMP9 in endothelial cells may allow this matricellular protein to regulate critical effectors of angiogenesis. The ECM and integrin binding capabilities of CTGF suggest a role for this molecule in the integration of angiogenic signals that may be crucial for orchestrating the shifting of signaling to different phases of angiogenesis and endothelial cell phenotype.

YAP1/SMAD1 binding enhances BMP signaling via target gene Id1 [71]. However, we were interested to discover if YAP1/SMAD1/5 binding was regulating Hippo pathway target genes. Recent studies have implicated endoglin in the regulation of CTGF expression, with a majority of these studies focused on CTGF’s pro-fibrotic nature. Interestingly, endoglin has been shown to both increase and decrease CTGF expression, depending on the context, which leads to a mechanism that is still poorly understood [98, 99]. In non-endothelial cells, BMP9 can activate p38MAPK [100, 101], which could potentially induce CCN expression [102]. However, we did not see significant induction of p38MAPK following BMP9 treatment (data not shown). Here we find that in endothelial cells endoglin expression is required for the expression of both CTGF and CYR61 in response to BMP9. Because CTGF and CYR61 have been shown to be involved in the remodeling and inflammatory response during angiogenesis, we hypothesize that the regulation of these secreted matricellular proteins plays a role in abnormal angiogenesis and AVM development. More work will be required to evaluate this hypothesis.

Interest in the importance of ECM formation and its regulation by endoglin and BMP9 led to the implication of key components in ECM composition. Recent studies have implicated the integrin α5β1 complex and its primary ligand, fibronectin, in the propagation of SMAD1/5 signaling via endoglin and ALK1 [103]. It was also shown that both the increase of integrin α5β1 expression and activation was TGFβ dependent and BMP9 independent. Our data suggest that an important aspect of BMP9 signaling is the regulation of the protein composition of sites of focal adhesion and endothelial cell-ECM interactions. This idea is supported by mouse studies using endoglin-null, fibronectin-null, and, most recently, YAP1-null mice, which are all embryonic lethal at day 9.5–10.5 due to vascular development defects [16, 17, 104–106]. The wound healing assays performed in these studies showed little difference in HUVEC’s ability to close the wound with or without BMP9 treatment when plated on fibronectin. We also looked at HUVEC response to BMP9 in transwell migration assays and, again, saw no significant change in response to BMP9 stimulation (data not shown). Other studies looking at endothelial cell migration in response to BMP9 have shown both pro- and anti-migration affects [23, 107–110]. This difference suggests that BMP9’s role in endothelial cell migration is context dependent and still poorly understood. Interestingly, wound-healing assays suggest an important role for endoglin in endothelial cell morphology during migration on ECM (fibronectin). Suppression of endoglin expression in HUVEC causes the cell morphology to be rounded and disoriented during wound closing compared to controls. This may be in part due to altered focal adhesion formation and YAP1/CTGF expression [73]. These data together suggest a mechanism whereby endoglin subcellular localization and signaling contributes to the quiescent state of the endothelial cell and promotes vessel maturation.

In the present studies, we identify molecular features of the adult lung of *eng* ^+/-^ mice that model the disease HHT1. A majority of the time, these mice are initially asymptomatic and develop a more evident HHT-characteristic phenotype as they age. In most mouse model studies, it has often been necessary to induce vascular injury in order to observe a response. Histological analysis of HHT1 patient AVMs, as well as *eng* ^+/-^ mouse lungs, has shown ECM remodeling defects around AVMs [111, 112]. Here, we show in the *eng* ^+/-^ mouse lungs an increase of ECM remodeling around the arteries as visualized by the HU177 antibody that identifies the expression of cryptic epitopes in collagen that are exposed during angiogenic events such as tumor development. A remodeling ECM is consistent with an increase in expression of MMP9 in the *eng* ^+/-^ mouse lungs. This observation suggests a more angiogenic state of the *eng* ^+/-^ mouse lung vasculature though other studies have shown endoglin deficiency to be anti-angiogenic. *In vitro* endothelial cell capillary formation assays [113], matrigel plug implants in the *eng* ^+/-^ mouse, [90] and endothelial and fibroblast cell tube formation assays [114] all indicate a decrease in angiogenesis in endoglin deficiency. However, these models all consist of ECM materials and *in vitro* environments that may not recapitulate *in vivo*. AVMs have been described as a result of abnormal angiogenesis and some of the most effective treatments for HHT symptoms are angiogenesis inhibitors [115, 116]. Our *in vitro* and *in vivo* experiments allow us to hypothesize an important role for endoglin localization and signaling to maintain a quiescent vasculature. The deficiency in endoglin expression in endothelial cells *in vitro* showed a cell with improper formation of focal adhesions, morphology, and migration compared to when endoglin expression is normal. *In vivo* analysis gives us an idea of how the endothelial cells are functioning and signaling in response to deficient endoglin levels in the lung.

The present study furthers recent research [18, 29] on endoglin dependent contributions to the inflammatory component of angiogenesis. Here we show BMP9 stimulation in endothelial cells represses the inflammatory chemokine axis of CCL2 and CCR2, which is accompanied by upregulation of CCL5 *in vitro*. Interesting recent studies indicate that in the presence of CCL2, the subcellular localization of MMP9-containing vesicles was altered. This relationship implicates BMP9 and endoglin in the regulation of ECM metabolism. *In vivo* studies to analyze the expression of CCL2 in the *eng* ^+/-^ and *eng* ^+/+^ mouse lungs suggest both an increase of CCL2 expression in the vasculature and the infiltrating inflammatory cells. Other studies in irradiated kidney of *eng* ^+/-^ and *eng* ^+/+^ mouse models suggest a decrease of inflammatory cytokine response including CCL2 [117]. In *eng* ^+/-^ and *eng* ^+/+^ mice injected with carrageenan or LPS to induce inflammation, there was a reduction in leukocyte infiltration of the lungs of the *eng* ^+/-^ mice when compared to the *eng* ^+/+^ [18]. The most recent studies implicate an impaired resolution of inflammation in the *eng* ^+/-^ mouse in response to chronic colitis [118]. The results of our experiments may differ from those in the preceding literature because our animals were uninjured and, subsequently, injury response mechanisms were not triggered.

CCR2 expression in endothelial cells contributes to CCL2-dependent macrophage transendothelial cell recruitment [119]. The CCL2/CCR2 signaling axis has recently been implicated in tumor development, suggesting endothelial expression of CCR2 to increase vascular permeability and responsiveness to CCL2 positive monocyte infiltration [120]. As well, both CCR2 and BMP9 signal transduction have been linked to the dysregulation of Notch signaling in endothelial cells and vascular remodeling [121, 122]. Taken together, our *in vitro* and *in vivo* results suggest a role for BMP9 and endoglin in regulated inflammatory chemokine signaling and the CCL2/CCR2 signaling axis. CCL5 appeared to be a target of BMP9, but relatively little is known about CCL5 elicited in endothelial cells. CCL5 enhances the binding of T cell subsets to endothelial cells [123] and expression of CCL5 on endothelial cells contributes to the formation of a permissive metastatic microenvironment, via a mechanism that involves selectin-mediated adhesion [124]. Finally, the CCL5/CCR5 and CCL2/CCR2 axes, along with SDF1/CXCR4, and E-Selectin regulation [29] may constitute dynamic BMP9-coordinated effectors of endothelial progenitor and stromal cell recruitment in wound healing [125].

In conclusion, it is important to note that while involvement of both zyxin and YAP1 in BMP9/ALK1/endoglin signaling strongly implicate HIPPO pathway crosstalk, further research will be required to elucidate whether other important points of crosstalk exist, and the extent to which Hippo-independent contributions by YAP1 to BMP9 signaling are of significance. Nonetheless, the present studies suggest a broad spectrum of activities that may be regulated by BMP9/Hippo crosstalk in endothelial cells. Additional studies are required for a fuller understanding of endoglin’s functions in endothelial cells.
